# Novel *KIF11* Mutation Associated with Microcephaly, Chorioretinopathy and Impaired Intellectual Development: 20 Years of Follow-Up

**DOI:** 10.3390/children12050560

**Published:** 2025-04-26

**Authors:** Ashley H. Yaskanich, Ami Patel, Monique Leys

**Affiliations:** Department of Ophthalmology and Visual Sciences, West Virginia University, Morgantown, WV 26506, USA; ami.patel@hsc.wvu.edu (A.P.); monique.leys@hsc.wvu.edu (M.L.)

**Keywords:** microcephaly with or without chorioretinopathy, lymphedema, impaired intellectual development, *KIF11* gene, familial exudative vitreoretinopathy, retinal detachment, scleral buckle, vitrectomy, visual rehabilitation

## Abstract

**Background:** *KIF11* mutations are responsible for a large portion of microcephaly with or without chorioretinopathy, lymphedema or impaired intellectual development (MCLMR). **Methods**: This report describes longitudinal ophthalmological management of an 8-year-old male pediatric patient presenting with MCLMR diagnosed in infancy and associated with a novel, de novo *KIF11* mutation. **Results**: The patient presented with ophthalmological features of low visual acuity and chorioretinal atrophy and later developed bilateral retinal detachments. Syndromic features included microcephaly and developmental delay. Scleral buckling and vitrectomy were ultimately performed in both eyes, with a period of conservative management in the interim. Postoperative visual acuity was preserved in the right eye, although poor in the left eye. The patient received low-vision rehabilitation services and was able to participate in school and extracurricular activities. **Conclusions**: Early recognition and close monitoring of ocular and systemic manifestations of *KIF11* mutations are important to optimize visual rehabilitation efforts.

## 1. Introduction

EG5, a kinesin protein product of the *KIF11* gene, is thought to play an important role in the division of neural stem cells, including the neural retina, and dysfunction in animal models leads to congenital anomalies [1]. *KIF11* mutations are thought to be responsible for all inherited, and nearly half of sporadic, cases of microcephaly with or without chorioretinopathy, lymphedema or impaired intellectual development (MCLMR; OMIM 152950) [2]. The prevalence of MCLMR is estimated to be less than 1 in 1,000,000 people worldwide [2,3]. We present a case of a patient with MCLMR and a novel, de novo *KIF11* mutation with longstanding follow-up at an academic eye center.

## 2. Case Presentation

**Figure 1:**
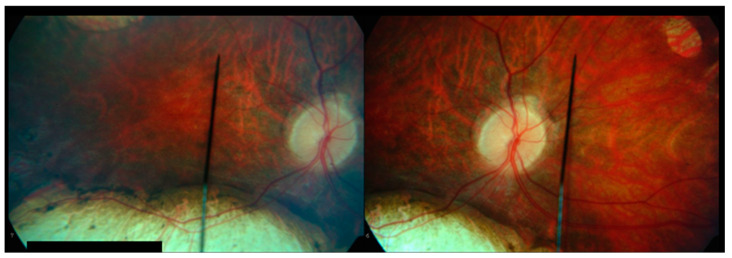
Chorioretinal atrophy of the right eye; macular view (**left**) and nasal retina (**right**), age 10.

**Figure 2:**
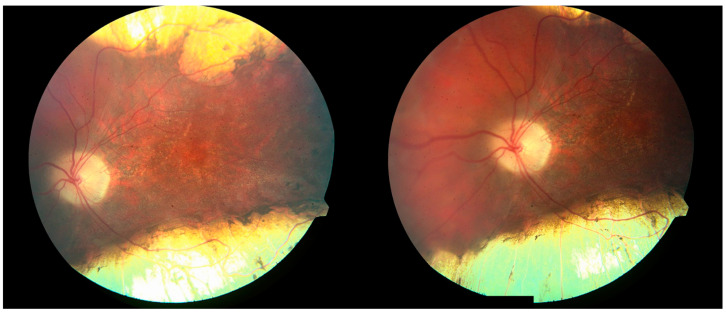
Chorioretinal atrophy of the left eye; macular view (**left**) and nasal retina (**right**), age 10.

An 8-year-old male presented to the pediatric ophthalmology clinic for evaluation of possible strabismus and poor vision. Past medical history was significant for microcephaly- and lymphedema-related developmental delays requiring special education and speech therapy, as well as a history of seizures (in remission without antiepileptic drugs for at least one year). The patient had been clinically diagnosed with microcephaly–lymphedema–chorioretinal dysplasia syndrome (MLCRD) prior to presentation. His height and weight were in the fifth percentile for his age. Family ocular history was positive only for strabismus, and there was a maternal family history of hereditary lymphedema. Upon initial examination, visual acuity was 20/400 eccentrically in the right eye with a correction of −0.50 +1.00 × 180 and 20/60 in the left eye with a correction of −0.50. Visual fields were full to confrontation, and extraocular movements were normal. The anterior segment examination was within normal limits. A dilated fundus exam demonstrated areas of retinal pigment epithelium deposits and chorioretinal atrophy (CRA) bilaterally (Figure 1 and Figure 2).

Electroretinography testing was performed with skin electrodes using LKC Utas user-defined protocol as described in the 2008 ISCEV standards, with a light-adapted background of 30 cd/m^2^ and flash intensity of 2.5 cd/s/m^2^ for both light-adapted and dark-adapted conditions [4]. ERG signals were abnormally low, even when multiplied by a maximum attenuation factor of 10, and responses were often delayed, including in light. These results suggested generalized retinal disease affecting both rods and cones. MRI of the brain and orbits demonstrated a parenchymal volume lower than expected for age, with prominent ventricles and sulci, possible periventricular white matter attenuation, and normal orbits. Dark adaptation was measured with the cone adaptation test and was found to be functionally normal. A mild red-green color deficiency was detected.

Through the Children’s Vision Rehabilitation Project (CVRP), an interdisciplinary outreach pediatric low-vision clinic in West Virginia with support of the Department of Education, the patient was prescribed a light-gathering magnifier for near objects and a 2.8× magnification monocular device for distance. The patient received additional support and therapy in school and was able to participate in farmwork and sports, during which he was instructed to wear polycarbonate safety glasses. He followed up in a retina clinic every six months thereafter, or more frequently as indicated by exams, imaging, or patient concerns.

By the age of 11, the patient had developed a macula-off retinal detachment (RD) in the left eye and a macula-on RD in the right eye. He underwent multiple surgeries including pars plana vitrectomy, scleral buckling, and cataract extraction with left intraocular lens placement. However, even after extensive intervention, the vision in his left eye gradually declined to no light perception by early adulthood. Given the patient’s monocular status and stability of the RD in the right eye, conservative management was pursued.

**Figure 3:**
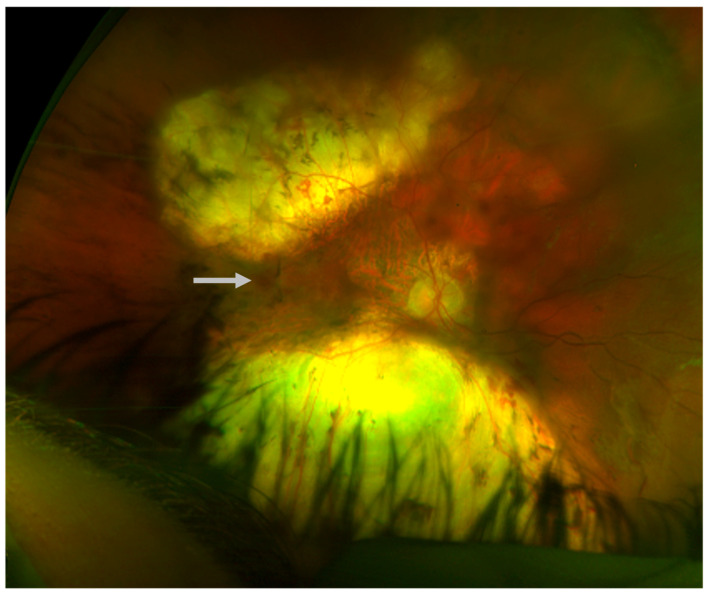
Vitreous hemorrhage (**arrow**), right eye, age 32.

**Figure 4:**
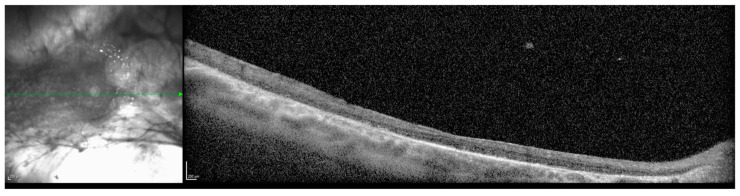
Optical coherence tomography (OCT) of macula, 8 months following pars plana vitrectomy with scleral buckle procedure, right eye, age 32. The vitreous hemorrhage had cleared, and the macula was flat. Resolution was poor due to nystagmus.

The patient continued to attend surveillance appointments for the next decade with a relatively stable clinical course. Next generation sequencing and Sanger sequencing of the *KIF11* gene and flanking introns performed in consultation with a medical geneticist revealed a previously undocumented, heterozygous de novo c.210+2del intronic variant of the *KIF11* gene (Prevention Genetics, 2017, Marshfield, WI, USA, NM_004523.3). The patient’s biological parents and two siblings declined clinical examination and genetic testing for the variant. At age 32, a small right vitreous hemorrhage was identified, and there was concern for progression and temporal foveal encroachment of the patient’s chronic RD (Figure 3). The patient underwent a right scleral buckle procedure with external drainage, limited pars plana vitrectomy, cryoretinopexy, and intravitreal injection of SF6 gas. Following postoperative recovery, visual acuity in the right eye improved to 20/200 from a baseline of 20/400, and the macula and fovea remained attached (Figure 4).

## 3. Discussion

Phenotypes from isolated Familial Exudative Vitreoretinopathy (FEVR) to systemic, syndromic presentations are attributable to *KIF11* mutations [5,6]. FEVR is an inherited disease characterized by poor vascularization of the peripheral retina, retinal neovascularization, subretinal exudates, and RD, and linked to multiple mutations in genes including *KIF11*, *FZD4*, *TSPAN12*, *LRP5*, *NDP*, *JAG1*, *ZNF408*, and *TUBGCP6* [7,8,9,10]. Cases of FEVR-like retinopathy have been documented with multiple syndromes, including 11q deletion syndrome, Turner syndrome, homocystinuria, CDMMR (chorioretinal dysplasia, microcephaly, and intellectual disability), MLCRD (microcephaly, primary lymphedema, and chorioretinal dysplasia), and other genetic conditions [9,10,11,12,13,14]. Due to the similarity of phenotypic and genotypic causes of MLCRD and CDMMR, these are now both considered to fall within the same classification; that is, MCLMR (OMIM 152950) [14]. MCLMR has an autosomal dominant inheritance pattern with incomplete penetrance, but as many as 40% of cases originate from de novo mutations [15,16]. *KIF11* mosaicism has been reported in asymptomatic parents of probands, complicating estimates of the frequency of these de novo mutations [17].

Here, MCLMR presented with bilateral RD, chorioretinal atrophy, microcephaly, developmental delay, and childhood seizures. Ocular features of MCLMR may include rod and cone dysfunction, chorioretinopathy, retinal folds, and RD [6,8,18]. Tractional RDs are especially associated with MCLMR, and amongst MCLMR patients, bilateral RDs are more highly associated with developmental delays [6,19]. The most common systemic features are microcephaly, learning disability, and lymphedema. In a subset of patients, epilepsy and cardiac defects are found [8,16,18].

In the present case, the patient underwent left scleral buckling and vitrectomy bilaterally, with an extended period of conservative management preceding surgery in the right eye. Following surgery, visual outcomes were poor in the left eye and slightly improved in the right eye. Though there is a dearth of studies on the management of ocular manifestations of *KIF11*-associated MCLMR specifically, numerous case series have documented FEVR management and outcomes. Intravitreal anti-vascular endothelial growth factor (VEGF) agents have been used with varying success [20,21,22]. Laser ablation may be used especially for earlier stages of FEVR with good visual outcomes [23,24]. As in this case, more advanced disease with RD necessitates surgical management, including scleral buckling, vitrectomy, and/or membrane peeling [23,25]. Anatomic reattachment is often successful, though repairs may be complicated and visual acuity outcomes are variable [24,26,27,28,29]. Lensectomy may also be performed in cases of anterior segment complications associated with FEVR to prevent secondary glaucoma [27,30]. Prognosis likely depends on disease stage and progression of fibrovascular proliferation at the time of surgery [23,24]. Here, the patient underwent biannual retinal exams during periods of relative stability and was followed more closely when necessitated by disease progression or acute events. The appropriate frequency of ophthalmological examinations in a patient with MCLMR may depend upon phenotypic severity at presentation and will likely vary over time.

The patient in this case received low-vision rehabilitation and assistive devices and was able to progress through schooling and participate in work and extracurricular activities. Visual rehabilitation is an important effort toward maximizing quality of life for patients affected by *KIF11*-assocated FEVR, MCLMR, and other vision-impairing conditions. Early recognition of ocular, neurologic, and genetic causes of vision loss and subtle behavioral indicators of vision difficulties in children allows for prompt referral to appropriate services. Rehabilitative goals change with the patient’s age; in a school-aged child as in the initial presentation here, emphasis is placed on assistance in the classroom with an individualized educational plan (IEP), which may include seating preference, magnification tools, and high-contrast electronic devices [31]. For physical education and extracurricular sports, protective lenses and other safety accommodations are recommended [31].

Numerous organizations exist to offer support, resources, and advocacy for patients with low vision and their families. A Germany-based nonprofit organization, KIF11 Kids e.V. (kif11kids.com; accessed on 8 March 2025), provides support and community specifically to families affected by *KIF11* mutations. Providers caring for pediatric patients with low vision should be familiar with these organizations.

## 4. Conclusions

Here, we document a longstanding follow-up of a case of MCLMR associated with a novel, de novo *KIF11* mutation presenting with microcephaly, developmental delay, CRA, and bilateral RD, for which both surgical and expectant management were utilized. Vision was preserved unilaterally. Physicians should be familiar with the ocular and systemic manifestations of *KIF11* mutations in children, as earlier retina specialist referral for close monitoring and intervention promotes visual rehabilitation.

## Data Availability

The original contributions presented in the study are included in the article, further inquiries can be directed to the corresponding author.

## Abbreviations

The following abbreviations are used in this manuscript:

MCLMR	Microcephaly with or without chorioretinopathy, lymphedema, or impaired intellectual development
RD	Retinal detachment
CRA	Chorioretinal atrophy
FEVR	Familial Exudative Vitreoretinopathy
